# Towards Understanding the Decomposition/Isomerism Channels of Stratospheric Bromine Species: *Ab Initio* and Quantum Topology Study

**DOI:** 10.3390/ijms16046783

**Published:** 2015-03-25

**Authors:** Saadullah G. Aziz, Abdulrahman O. Alyoubi, Shaaban A. Elroby, Osman I. Osman, Rifaat H. Hilal

**Affiliations:** 1 Chemistry Department, Faculty of Science, King Abdulaziz University, Jeddah B.O. 208203, Saudi Arabia; E-Mails: saziz@kau.edu.sa (S.G.A.); aalyoubi@kau.edu.sa (A.O.A.); skamel@kau.edu.sa (S.A.E.); oabdelkarim@kau.edu.sa (O.I.O.); 2 Chemistry Department, Faculty of Science, Beni Suef University, Beni Suef 6251, Egypt; 3 Chemistry Department, Faculty of Science, Cairo University, Cairo 12613, Egypt

**Keywords:** bromine bonds, quantum topology, NBO analysis, DFT calculations, stratospheric bromine species

## Abstract

The present study aims at a fundamental understanding of bonding characteristics of the C–Br and O–Br bonds. The target molecular systems are the isomeric CH_3_OBr/BrCH_2_OH system and their decomposition products. Calculations of geometries and frequencies at different density functional theory (DFT) and Hartree–Fock/Møller–Plesset (HF/MP2) levels have been performed. Results have been assessed and evaluated against those obtained at the coupled cluster single-double (Triplet) (CCSD(T)) level of theory. The characteristics of the C–Br and O–Br bonds have been identified via analysis of the electrostatic potential, natural bond orbital (NBO), and quantum theory of atoms in molecules (QTAIM). Analysis of the electrostatic potential (ESP) maps enabled the quantitative characterization of the Br σ-holes. Its magnitude seems very sensitive to the environment and the charge accumulated in the adjacent centers. Some quantum topological parameters, namely ∇^2^ρ, ellipticity at bond critical points and the Laplacian bond order, were computed and discussed. The potential energy function for internal rotation has been computed and Fourier transformed to characterize the conformational preferences and origin of the barriers. NBO energetic components for rotation about the C–Br and O–Br bonds as a function of torsion angle have been computed and displayed.

## 1. Introduction

The important role of bromine species in ozone depletion [1,2,3,4] in the Antarctic spring caused much research on these species in polar regions. It is assumed that the implication of stratospheric bromine species is analogues to that of the chlorinated species [2,3,5]. Furthermore, bromine species seem to play a potential role on ozone depletion in the marine boundary layer [6]. The reaction between bromine oxides with HOx species leading to the production of methyl bromide is of particular importance in this respect [7,8,9]. This process becomes more efficient in regions with high OH concentration. Furthermore, oxybromides which absorb light at longer wavelengths, will certainly contribute to ozone depletion.

The electronic absorption spectrum, in the gas phase, of CH_3_OBr has been reported in the 230 < λ < 400 nm range [10,11]. However, very little is known about the energetics of the photochemical/photophysical dissociation and isomerisation of CH_3_OBr. In order to evaluate the role of bromine species, in general and that of CH_3_OBr in particular, in ozone depletion, knowledge of the heat of formation and the electronic characteristics of the bromine bonds are required. The isomeric system CH_3_OBr/CH_3_BrO/BrCH_2_OH is suggested to play the major role in these photochemical processes, yet no systematic study of the energies, structures and dynamics of the excited states has been published.

Quantum mechanical calculations have been carried out to study chemical bonding [12,13,14] and possible decomposition pathways of methyl hypobromite CH_3_OBr [15] in comparison with the other two similar systems, CH_3_OF and CH_3_OCl [16]. It was shown that decomposition of bromoethanol proceeds by a 1,2 HBr elimination process [17]. The photofragmentation processes of BrCH_2_OH have been studied using *ab initio* multi-reference configuration interaction calculations for ground and excited states [12]. Several different calculations have also been carried out to examine the decomposition mechanism of the chloro and floro analogues [18,19,20].

**Figure 1:**
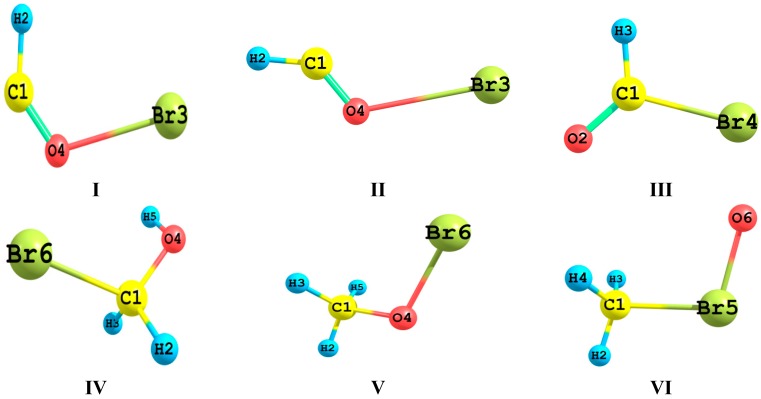
The optimized geometric structure and atom labeling of *cis*-BrOCH (**I**); *trans*-BrOCH (**II**); BrCHO (**III**); BrCH_2_OH (**IV**); CH_3_OBr (**V**); and CH_3_BrO (**VI**).

The ultimate goal of the present research project is to investigate the decomposition/isomerisation dynamics of the isomeric system CH_3_OBr/CH_3_BrO/BrCH_2_OH. Several different decomposition and/or isomerization channels have been proposed including the HCOBr (*cis*)/*trans* system. The first step to achieve this goal, which is addressed in the present communication, aims at a fundamental understanding of bonding characteristics of the C–Br and O–Br bonds. The target molecular systems are the isomeric CH_3_OBr/BrCH_2_OH system and their decomposition products which are suggested to play a major role in ozone depletion processes. We are interested in evaluating the significance and relative importance of stereoelectronic effects in these systems. The characteristics of the C–Br and O–Br bonds will be theoretically explored and analyzed via natural bond orbital (NBO), quantum theory of atom in molecules (QTAIM) and electrostatic potential analyses. Figure 1 displays the bromine-containing species considered in the present work.

## 2. Results and Discussion

### 2.1. Relative Stabilities of Isomeric Forms

The appropriate theoretical model should result in as accurate and flexible wave functions as possible and should also be cost-effective, bearing in mind that these wave functions will ultimately be used in very demanding computations of the on-the-fly dynamics for the decomposition/isomerization of the bromine-containing species in their excited states. To achieve this goal, we have carried out sophisticated calculations of the geometries and frequencies at the couple-cluster single-double (triple) CCSD(T)/6-311++G** level of theory. These very expensive calculations have been used as reference for the more economic models. Calculations at the different density functional theory (DFT) levels and at the HF-MP2 level of theory are compared to the CCSD(T) results in Table S1 of the supplementary material. Four different basis sets have also been examined in the present case. Comparison of the geometries and energies calculated using different basis sets is presented in Table S2 of the supplementary data. Careful inspection of the data in these tables reveals that acceptable level of accuracy and flexibility may be achieved at the B3LYP/aug-cc-pVTZ level of theory. In fact, this level of theory is able to produce geometries and electron density distributions of the same quality as that of the CCSD(T) level at a much reduced cost. This level will be adopted in all the calculations presented in the present communication.

Furthermore, in addition to assessing the appropriate theoretical model detailed in the aforementioned paragraph, we carried out further exhaustive calculations to determine accurate energy values to be used in thermochemical analysis of the studied systems. In such calculations, we attempt to reach complete basis set limit. Table 1 presents the total energies of the studied isomeric systems computed at the CCSD(T)/aug-cc-pVXZ (X = 2, 3, 4) level of theory. Energies extrapolated to the CBS are also given in Table 1. For the isomeric system CH_3_OBr/CH_3_BrO/BrCH_2_OH, the alcohol form is more stable than CH_3_OBr and its hypervalent bromine isomer by 35.12 and 75.25 kcal/mol, respectively. For the isomeric system HCOBr(*cis*)/HCOBr(*trans*)/BrCHO, the aldehyde form is much more stable than the HCOBr(*cis*) and its trans form by 67.11 and 75.89 kcal/mol, respectively. Increasing the quality of the basis set (mainly polarization functions) from double zeta (DZ) to quadruple-zeta (QZ) increases the relative energies by 0.28 to 0.48 atomic unit (au). Most of this energy stabilization (79%) is due to the increase in quality in going from DZ to triple-zeta (TZ), the other 21% may be gained at a much higher cost on going to QZ. In order to evaluate the effect of the diffuse functions on the computed energies we have carried out similar CCSD(T) calculations using the cc-pVTZ basis set. Results are also included in Table 1 as numbers in parentheses. Inclusion of diffuse functions seems to lower the computed energies by about 0.02 au except for the case of the hypervalent bromine isomer CH_3_BrO where diffuse functions seems to be much more effective where its effect exceeds 0.09 au.

**Table 1:** CCSD(T) total energies (au) computed using different basis sets for the studied isomer bromine containing systems.

Model	I	II	III	IV	V	VI
aug-cc-pVDZ	−2686.10959	−2686.09582	−2687.40968	−2687.40968	−2687.35216	−2687.27657
aug-cc-PVTZ	−2686.37221 (−2686.35370)	−2686.35838 (−2686.34045)	−2687.68607 (−2686.45778)	−2687.68607 (−2687.66428)	−2687.62962 (−2687.60696)	−26875.62997 (−2687.53326)
aug-cc-pVQZ	−2686.47047	−2686.45571	−26877.86817	−2687.78682	−2687.72951	−2687.66377
CBS	−2686.50733	−2686.49332	−2686.61433	−2687.82633	−2687.77034	−2687.70635

### 2.2. Structures and Vibrational Frequencies

In the present section, we will attempt to localize and identify the structural characteristics and energetic of the C–Br and O–Br bonding interactions. Figure 1 presented the optimized geometries of the bromine-containing species which will be the subject of the present study.

Let us start by studying the geometric features of the isomeric system CH_3_OBr/CH_3_BrO/BrCH_2_OH. The alcohol form BrCH_2_OH is much more stable by 33.9 and 106.2 kcal/mol than CH_3_OBr and its hypervalent bromine isomer CH_3_BrO isomer, respectively. The three isomers possess staggered structures with *Cs* symmetry.

**Figure 2:**
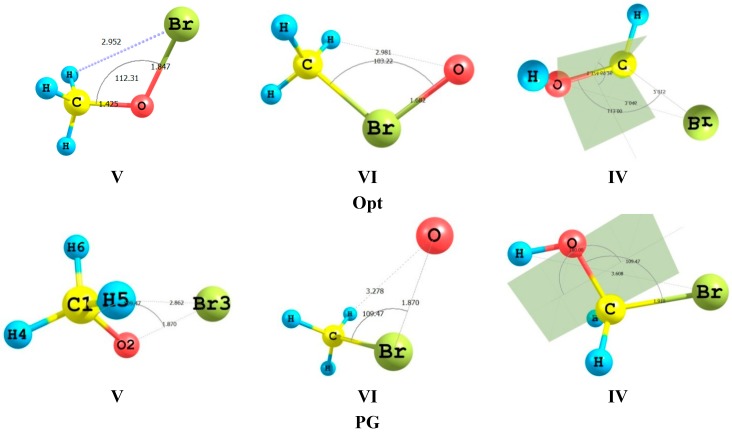
Comparison of the geometric features of the idealized PG and DFT optimized structure (Opt) for the isomeric system CH_3_OBr/CH_3_BrO/BrCH_2_OH.

The geometry differences are associated and focus on methyl torsions; the associated potential energy functions often exhibit significant skeletal flexing perturbations (couplings to stretching and bonding degrees of freedom) that modify an idealized rigid-rotor picture. Figure 2 compares the geometric features of the rigid-rotor limit of idealized Pople–Gordon (PG) geometry (that is localized geometry in the valence bond natural bond orbital (NBO) basis) [21] and DFT/B3LYP/aug-cc-pVTZ optimized structures in the delocalized Molecular orbital (MO) basis for the isomeric system CH_3_OBr/CH_3_BrO/BrCH_2_OH. The optimized geometries deviate strikingly from idealized PG form particularly with respect to Br…H separation and the dihedral angle specifically in case of BrCH_2_OH. For CH_3_OBr the optimized C–O–Br angle open significantly 112.5° thereby partially relieve the apparent steric congestion. This is a result of the mutual repulsion of the lone pair electrons localized on both the Br and O atoms. The isomeric form, CH_3_BrO is also enjoying a staggered conformation with the C–Br–O angle much smaller *ca.* 103°. This smaller value of the C–Br–O angle suggests that there is a π-overlap between the lone-pair electrons of the bromine atom and that of the oxygen atom. This π-overlap imparts partial double bond character to the Br–O bond leading to decrease in its length to ~1.7 Å with the consequent decrease in electron repulsion in the carbon–bromine bond region. This point may be further clarified by NBO analysis of the total self-consistent field (SCF), deletion and delocalization energies of the tautomeric system. In these calculations, all the Rydberg and antibond orbitals, which are the non-Lewis NBO orbitals, that usually appear in the NBO analysis are deleted. The result of this deletion is the energy of the idealized NBO natural Lewis structure, with all Lewis NBOs doubly occupied. Unlike other deletions, in which there is a slight loss of variational self-consistency due to the redistributed occupancy of the deleted orbitals, the result of this deletion corresponds rigorously to the variational expectation value of the determinant of doubly occupied Lewis NBOs. This analysis is presented in Table 2. For the tautomeric system IV, V and VI, IV is the most stable by 34.742 and 77.295 kcal/mol compared to V and VI, respectively. In an ideal Lewis Structure, V is more stable than IV and VI by 23.947 and 75.054 kcal/mol, respectively. The competitiveness of IV is due to the strong delocalization gaining 140.285 kcal/mol. The geometric features of IV reveal the origin of its competitiveness. Thus, the C–Br bond is now longer than 2.0 Å while the C–O bond approaches a double bond length with a value of 1.37 Å. The O–C–Br bond angle is 112°. The interaction in the C–O bond region is markedly increased at the expense of that in the C–Br. However, the repulsive interaction involving the lone pair electrons residing on the Br atom causes the observed enlargement of the Br–C–O angle.

**Table 2:** NBO analyses of the total SCF energy (au), deletion (au) and delocalization (kcal/mol) energies of the studied bromine-containing species computed at the B3LYP/aug-cc-pVTZ level of theory.

Substrate	Total SCF Energy	Deletion Energy	Delocalization Energy	Rotation Barrier, kcal/mol
I	−2688.09805	−2682.98976	3205.503	
II	−2688.08126	−2683.11274	3117.798	
III	−2688.19984	−2687.94231	161.600	
IV	−2689.41115	−2689.18759	140.285	42.406
V	−2689.35579	−2689.22576	81.596	2.749
VI	−2689.28797	−2689.10615	114.097	0.763

The basic issue is whether the stabilizing interactions in this tautomeric system should be primarily regarded as decrease of “steric repulsion” or increase of “bonding attraction”. Direct calculation of the potential energy function for rotation around the C–Br and the O–Br bonds and its Fourier transform analysis can be brought to bear on this issue.

**Figure 3:**
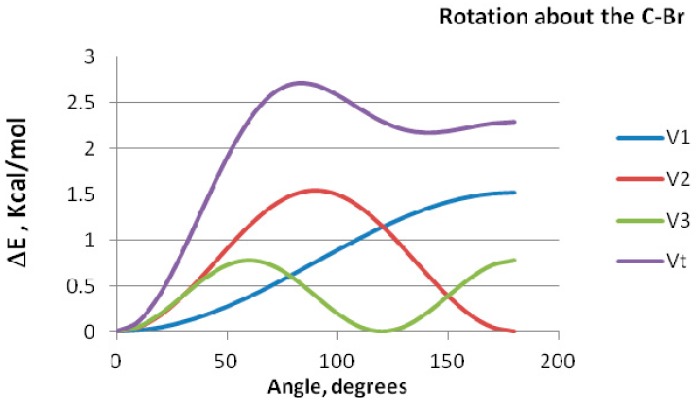
Fourier decomposition of the potential function for rotation about the C–Br bond in CH_3_BrO.

The computed rotation barriers are depicted in Table 2. The large difference in magnitude between the energy barriers for BrCH_2_OH and that of the CH_3_OBr and CH_3_BrO indicate clearly that rotation about C–Br and O–Br bonds is of a completely different nature than that about the C–O bond. Delocalization interaction underlies the large rotation barrier for rotation about the C–O bond in BrCH_2_OH. The origin of this rotational barrier can be identified by Fourier analysis of internal rotation function as detailed by Pople *et al.* [22,23]. In this analysis, the potential energy is partitioned into components, namely bond dipoles, conjugative and electrostatic repulsion contributions The Fourier transform equation can be represented as V(φ) = V_1_(φ) (1 − cos φ) + V_2_(φ) (1 − cos2φ) + V_3_(φ) (1 − cos3φ), where φ is the rotation angle, the V_1_ represents the interaction of bond dipoles, V_2_ represents the conjugative interaction, and V_3_ is an electrostatic bond–bond repulsion term. Fourier decomposition of the potential function for CH_3_BrO has been performed; results are displayed in Figure 3.

All three components of the Fourier transform contribute significantly to the potential barrier. Thus, the one-fold term shows maximum repulsion between local dipoles at φ = 180°, and maximum attraction at φ = 0°. Conjugative stabilization, as reflected by the V_2_ term, is maximum at φ = 0°and 180°. The three fold electrostatic interaction component shows maximum stabilization at φ = 0° and 120° and is repulsive otherwise. The potential energy function of rotation for CH_3_OBr shows almost the same trend.

HCOBr is one of the decomposition products of the isomeric system CH_3_OBr/CH_3_BrO, which is believed to play a central role in O_3_ depletion in the Arctic troposphere. This species can exit in a *cis*(I) or a *trans*(II) conformations. It should be mentioned that the optimization of this species always falls into the *cis*-conformation, which seems to be the global minimum structure on the potential energy hypersurface. The *trans*-conformation can only be obtained by freezing the dihedral angle during full optimization. The cis form (I) is more stable by 10.825 kcal/mol. The geometric features of this species shows the longest C–Br bond length of an average value of 2.5 Å, reflecting a minimal attractive interaction of the *d*-orbitals of Br– with the *p*-orbitals of carbon. The C–O bond length shows its minimum value in this species which reflects a tight binding and pronounced double bond character. This is further confirmed by a C–O–Br bond angle approaching 120°, a typical *sp*^2^ hybridization scheme for the oxygen atom. The isoelectronic compound BrCHO (bromo formaldehyde) (III) shows the same bonding characteristics as the HCOBr species. Comparison of the NBO results for the total SCF, deletion and delocalization energies of the tautomeric system I, II and III is presented in Table 2. It is apparent that the structure III is more stable than I and II by 63.877 and 74.410 kcal/mol, respectively. In a hypothetical Lewis structure, I is slightly more stable than III. The competitiveness of I and II are brought about by extensive hyperconjugation that gained them 3205.503 and 3117.798 kcal/mol, respectively.

Table 3 displays the vibration frequencies and intensities of the relevant Br–O and C–Br bonds and angles. For comparison, frequencies reported in the literature [15,20] are included in the same table. Data in Table 3 reveal that there are two types of C–O bonds in the studied compounds. C=O bonds vibrate at higher frequencies (1820–1950 cm^−1^ range) with much higher intensities than that of the C–O stretching vibrations which appear at lower frequencies (in the 1050–1100 cm^−1^ range). The correspondence with the literature values are satisfactory bearing in mind that our frequency calculations are carried out within the harmonic approximation. In order to evaluate and assess the importance of anharmonic corrections of vibration modes, calculations have been performed taking in account the anharmonicity of the vibrations. The calculations were carried out in the gas phase using the VPT2 method as implemented by Barone [24,25] in the Gaussian program package [26]. In all cases, the finest DFT integration grid was selected by using SCF = tight in the command line. Results are included in Table 3. As a general trend, the anharmonic vibration frequencies are lower than the corresponding harmonic frequencies by few frequency units. There is also a clear but small effect on the computed anharmonic intensities. Furthermore, attachment of a Br atom to the carbon of a carbonyl group causes a blue shift and lowering of the intensity of the C–O stretching vibration. The presence of a C=O group shifts considerably the electron density away from the O–Br or the C–Br bond regions with a consequent blue shift of the O–Br and the C–Br stretching frequencies. Thus, in case of BrOCH the Br–O stretching mode appear at a very low frequency of 160–196 cm^−1^ as compared to 620–730 cm^−1^ range for this mode in case of CH_3_OBr, its tautomer CH_3_BrO and isomer BrCH_2_OH. Calculation of the vibration spectrum of the OBr radical predicted the stretching vibration mode of the O–Br bond at 742 cm^−1^. In case of the CH_3_BrO where a methyl group is attached to the bromine, ν_Br–O_ shifts but slightly down field where as such a shift became more pronounced in case of the tautomeric compound CH_3_OBr, where ν_Br–O_ appears at 625 cm^−1^.

**Table 3:** Vibrational Frequencies and Intensities (km/mol) for the studied bromine-containing compounds.

Species	ν, cm^−1^	Relative Intensity	ν_anharmonic_, cm^−1^	Relative Intensity (Anharmonic)	Assignment
*cis*-BrOCH (I)	1924.6 (2068) ^a^	601.1	1905.218	613	C–O str.
	196.8 (206) ^a^	65.3	197.912	59	O–Br str.
	292.2 (348) ^a^	13.0	293.888	13	Br–O–C angle bending
*trans*-BrOCH (II)	1979.7	588.5	1957.464	590	C–O str.
	161.6	71.44	157.284	62	O–Br str.
	253.8	86.2	221.901	97	Br–O–C angle bending
BrCHO (III)	1851.0 (1799) ^a^	466	1836.751	484	C–O str.
	633.1 (663) ^a^	168	626.148	172	C–Br str.
	351.5 (370) ^a^	13	350.071	14	O–C–Br angle bending
BrCH_2_OH (IV)	1102.7 (1126) ^a^	286.1	1069.0	299	C–O str.
	560.5 (625) ^a^	107	549.1	129	C–Br str.
	284.0 (306) ^a^	37	290.0	22	O–C–Br angle bendin
CH_3_OBr (V)	1054.4 (1048) ^a^	63.2	1466.6	11	C–O str.
	998,1 (581) ^a^	40	967.0	38	O–Br str.
	307.1 (319) ^a^	3	303.5	4	C–O–Br angle bending
CH_3_BrO (VI)	657.3 (723) ^b^	40.1	648.6	31	Br–O str.
	516.7 (530) ^b^	1.9	505.6	2	C–Br str.
	207.7 (222) ^b^	5.5	208.0	6	C–Br–O angle bending

^a^ reference [15]; ^b^ reference [20].

### 2.3. NBO-Based Quantification of Stereoelectronic Interactions

Table 4 lists the second order perturbation estimates of the hyperconjugative energies of I, II and III. The factors that contribute to the total stabilities of tautomers include the steric hindrance, electrostatic repulsion and hyperconjugation. As shown in Figure 1, the steric hindrance and electrostatic repulsion are minimal in III compared to II and I. These two factors have led to the overall stability of III compared to I and II by 63.877 and 74.410 kcal/mol, respectively. It is clear that I experiences the maximum steric effect with comparable electrostatic repulsion compared to II. Despite this, I is more stable than II by 10.533 kcal/mol.

The competitiveness of I compared to II is due to the strong n_2O4_→σ*_C1–Br3_, σ*_C1–Br3_→σ*_C1–O4_, σ*_C1–Br3_→n_2O4_ and n_1O4_→n_2O4_ interactions that yielded 187.75, 11.16, 13.13 and 15.38 kcal/mol for the former and 195.27, 4.81, 6.93 and 1.23 kcal/mol for the later. It is clear that although the n_2O4_→σ*_C1__–Br3_ interaction favours II by 7.52 kcal/mol; the later three interactions favour I by 26.70 kcal/mol.

As shown in Table 4, II has been favoured by 77.172 kcal/mol due to the minimal steric hindrance, while I gained 87.704 kcal/mol compared to II as a result of the strong delocalization. That is, the overall preference of I of 10.532 kcal/mol is mainly due to hyperconjugation. Tables S3 and S4 of the supplementary data show the details of preference of III compared to I (63.877 kcal/mol) and II (74.410 kcal/mol). This means that I, II and III have fairly comparable total energies. I and II have bridged the energy gap through extensive hyperconjugation.

**Table 4:** Second order perturbation (E^(2)^) estimation of the hyperconjugative energies (kcal/mol) of *cis*-BrOCH (I), *trans*-BrOCH (II), and BrCHO (III) which were calculated using B3LYP/aug-cc-pVTZ level of theory.

Interaction	I	II	III
σ_C1–H2_→n_2O4_	6.58	9.74	–
σ_C1–H2_→σ*_C1–Br3_	7.71	8.27	3.52
σ_C1–Br3_→σ*_C1–H2_	3.82	4.18	1.90
σ*_C1–Br3_→σ*_C1–O4_	11.16	4.81	–
σ*_C1–Br3_→σ_C1–Br3_	8.30	7.72	1.76
σ*_C1–Br3_→n_2O4_	13.13	6.93	–
n_1O4_→n_2O4_	15.38	1.23	–
n_1O4_→σ*_C1–H2_	2.01	–	1.32
n_1O4_→σ*_C1–Br3_	4.02	–	–
n_2O4_→σ*_C1–H2_	5.69	7.92	20.31
n_2O4_→σ*_C1–Br3_	3.17	12.06	52.67
n_2O4_→σ*_C1–Br3_	187.75	195.27	–
n_2O4_→σ*_C1–O4_	5.25	13.14	–
n_2Br3_→σ*_C1–Br3_	2.52	3.03	–
n_2Br3_→σ*_C1–O4_	–	–	5.03
n_3Br3_→π_C1–O4_	–	–	16.55
Total	276.50	274.30	103.06

Table 5 depicts the second order perturbation estimates of the hyperconjugative energies of IV, V and VI. NBO analyses of the total SCF, deletion and delocalization energies (a.u.) of IV, V and VI are listed in Tables S5–S8 of the supplementary data. It is clear that IV is more stable than V and VI by 34.741 and 77.295 kcal/mol, respectively. In an ideal Lewis Structure V becomes the most stable followed by IV despite the electrostatic repulsion between the negatively charged carbon and oxygen atoms. This is perhaps due to the proximity of the two Br and CH_3_ bulky groups in both IV and VI. It is worth mentioning that V is more stable than VI by 42.553 kcal/mol. Not just that, also the ideal Lewis Structure of V is more stable than that of VI by 75.054 kcal/mol.

This stems from the strong steric hindrance between the two bulky CH_3_ and Br groups and the ineffective electrostatic repulsion between the negatively charged carbon and oxygen atoms and the attraction between the negatively charged carbon atom and positively charged bromine atom. However, the total energies of the two tautomers are comparable. The competitiveness of VI is due to hyperconjugation that yielded 32.501 kcal/mol compared to that of V.

The most influential hyperconjugative interactions (*cf*. Table 5) (n_2O4_)n_2Br5_→σ*_C1–Br6_, (n_2O4_)n_2Br5_→σ*_C1–H3_(σ*_C1–H5_), (n_2Br6_)n_3O6_→σ*_C1–Br5_(σ*_C1–O4_) and n_3Br6_→σ*_C1–O4_, have contributed 21.09, 3.57, ˂0.5 and 5.25 kcal/mol respectively for IV; 5.61, 5.61, 1.48 and 1.61 kcal/mol respectively for V and 2.30, 2.19, ˂0.5 and 15.83 kcal/mol, respectively for VI. Therefore the origin of preference of IV is mainly due to the n_2O4_→σ*_C1–Br6_ and n_3Br6_→σ*_C1–O4_ donor–acceptor interactions; while the vicinal antiperiplanar σ_C1–H2_→σ*_O4–Br6_ and lone pairs-antibonding n_2O4_→σ*_C1–H3_ and n_2O4_→σ*_C1–H5_ interactions contribute mostly in V. However, the most influential hyperconjugation of VI was a lone pair donor and antibonding bond acceptor (n_3O6_→σ*_C1–Br5_).

**Table 5:** Second order perturbation (E^(2)^) estimation of the hyperconjugative energies (kcal/mol) of BrCH_2_OH(IV), CH_3_OBr (V), and CH_3_BrO(VI) which were calculated using B3LYP/aug-cc-pVTZ level of theory.

Interaction	IV	V	VI
σ_C1–H2_→σ*_O4–H5_ (σ*_O4–Br6_)(σ*_Br5–O6_)	3.09	6.63	1.42
σ_O4–H5_(σ_O4–Br6_)→σ*_C1–H2_	2.16	2.25	–
σ_C1–H2_→σ*_C1–Br5_	–	–	1.63
n_1O4_→σ*_C1–H2_	2.87	1.61	–
σ_C1–H3_→σ*_C1–Br5_	–	–	1.35
σ_C1–H4_→σ*_C1–Br5_	–	–	1.36
(n_2O4_)n_2Br5_→σ*_C1–Br6_(σ*_C1–H3_)(σ*_C1–H4_)	21.09	5.61	2.30
(n_2O4_)n_2Br5_→σ*_C1–H3_(σ*_C1–H5_)	3.57	5.61	2.19
n_1O4_→σ*_C1–H3_	2.95	–	–
(n_2Br6_)n_3O6_→σ*_C1–Br5_(σ*_C1–O4_)	–	1.48	15.83
n_3Br6_→σ*_C1–O4_	5.25	1.61	–
σ_C1–Br5_→σ*_Br5–O6_	–	–	1.11
n_2Br6_→σ*_C1–H2_	1.55	–	–
n_2Br6_→σ*_C1–H3_	1.71	–	–
Total	44.24	24.80	27.19

**Figure 4:**
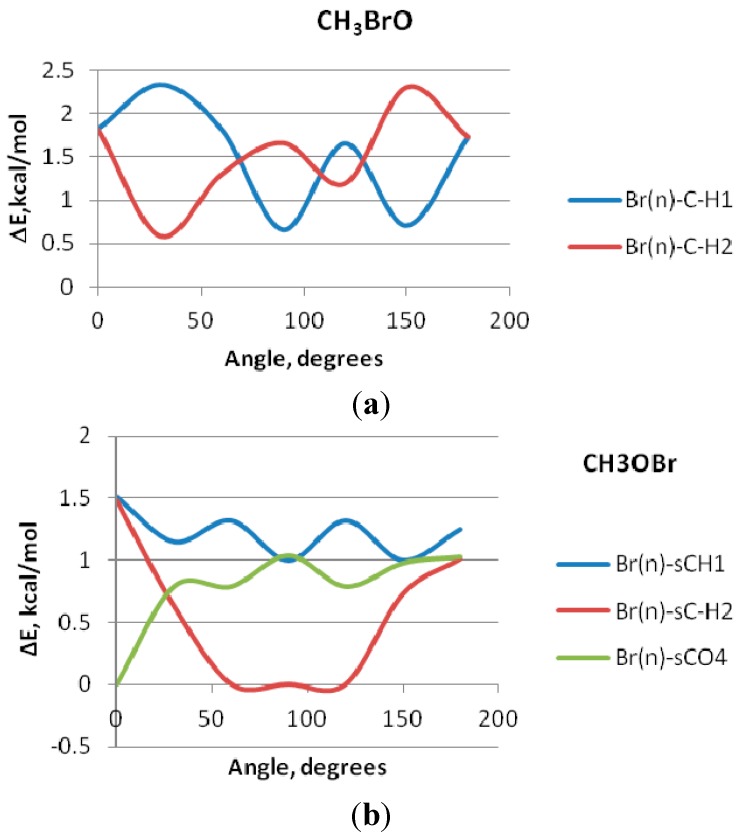
Variation of the leading NBO donor–acceptor interaction energies with the torsion angle of rotation about (**a**) O–Br and (**b**) C–Br bonds.

The C–F bond has been shown [27] to impact the conformational preferences of organic compounds suggesting its potential utility as a molecular design tool. This impact of the conformational preferences is due to stereoelectronic effects, specifically the anomeric [28] and gaughe effects [29]. These effects may be defined as the hyperconjugative interaction of the fluorine lone pair with the σ framework. There is no corresponding studies on the C–Br bonds. It is thus interesting to examine, quantitatively, (1) whether such effects exists in the case of the C–Br and O–Br bonds; and (2) the extent to which it impacts the conformational preference of the studied compounds. To that end the interactions of the bromine lone pair (nBr) with the σ* C–H MO have been computed as a function of torsion angle of rotation about the C–Br and O–Br bonds. Results are displayed in Figure 4a,b.

Figure 4 displays the calculated NBO energetic components for rotation about the C–Br and O–Br bonds as a function of torsion angle. Each of the two figures displays torsion variation of hyperconjugative interactions of the Br lone pairs n(2) and n(3) with the σ*C–H MO’s. These are the leading donor–acceptor contributions arising from vicinal hyperconjugative interactions with neighboring sigma-bond. In case of CH_3_OBr an additional donor–acceptor interaction term results from the Br(n) interaction with the σ* acceptor orbitals of the C–O NBO. These interaction curves bear similarity to the potential function for rotation displayed in Figure 2. It is clear from the similar vicinal environment in each of the two cases studied; the hyperconjugative details of methyl rotation are seen to be very similar. In case of CH_3_BrO, the two Br(n)-σ*(C–H) interactions work in a reciprocal way but their effects are cooperative, in general. For the CH_3_OBr case, the Br(n)-σ*CH and the Br(n)-σ*CO work cooperatively in a periodic manner, whereas, the second Br(n)-σ*CH interaction behave differently. It has a destabilizing effect in the 60°–120° region.

### 2.4. Electrostatic Potentials and Quantum Chemical Topology Analyses

**Figure 5:**
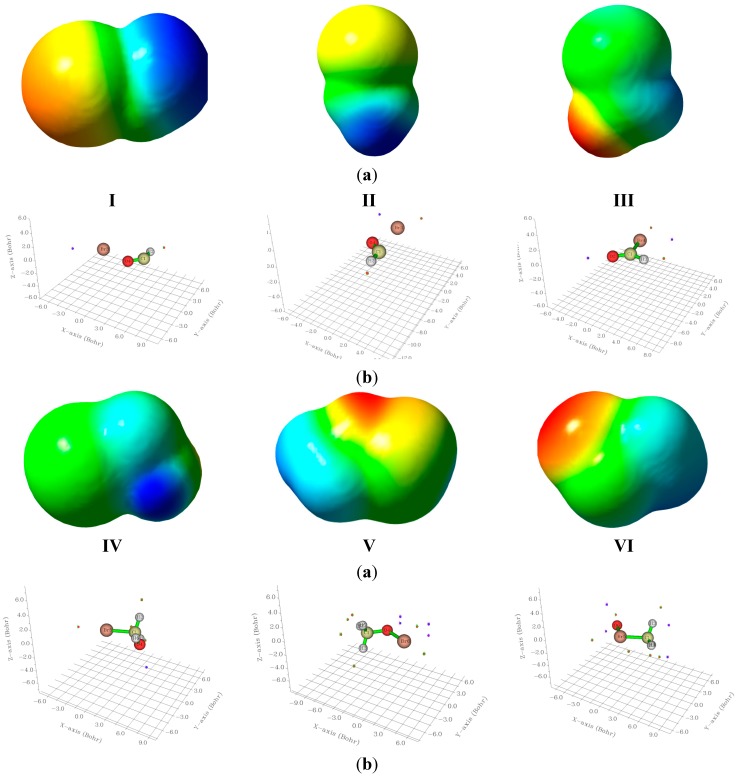
(**a**) Electrostatic potentials on the 0.001 au electron density surface; and (**b**) three dimensional graphs indicating the most positive maxima (red) and most negative minima (blue) surface points, for compounds **I**–**VI**.

The 3D electrostatic potential maps for the studied bromine species, are computed and visualized using GaussView 5.0 [30] and are displayed in Figure 5. The location of the surface maxima (red) and surface minima (blue) are computed and visualized using the Multiwfn software package [31,32]. In this software package the default isovalue is 0.001 au. The points so computed are illustrated in a 3D graphs in the same figure. The magnitudes of the most positive (V_S,max_) and most negative (V_S,min_), surface points are given in Table 6. In all molecular species studied there exists a surface maximum (positive hole) along the C–Br and the O–Br bonds at the end region of the bromine atom. In all cases, these surface maxima are surrounded by an electroneutral region and surface local minimum point alongside the X–Br bond near the Br atom (*cf*. Figure 5). Such a bromine positive region is referred to as the σ-hole, and seems to be a characteristic of all C–halogen bonds [33,34,35].

The bromine σ-hole in BrCH_2_OH (IV) shows its smallest value. This is most probably due to the large positive electrostatic potential of 78.902 kcal/mol surrounding the carbon atom. The Br σ-hole in BrCHO(III) is almost double that in the case of I although the bonding situations seem similar. In II, the positive potential is concentrated on H rather than the carbon atom. The isomeric system CH_3_OBr/CH_3_BrO shows also different trends for both the σ-holes and the surface minima around the oxygen atoms. Thus, the hypervalent bromine shows a smaller σ-hole and almost double negative potential at the oxygen atom. These represent global minimum on the surface, its large negative value is owing to the lone pair of oxygen. In case of BrOCH (*cis*)(I) the σ-hole is huge (107.88 kcal/mol) in contrast to its trans isomer (II) which shows a much smaller value.

Table 6 also shows some topological properties for the studied bromine species computed at the QTAIM level of theory. The theoretical basis of the theory has been detailed before and will not be repeated here [36]. The Laplacian of the electron density at the bond critical points (BCP) of the isomeric system CH_3_OBr/CH_3_BrO/BrCH_2_OH are all negative indicating accumulation of charge density in the X–Br bond region. However, their magnitudes vary considerably. Thus, while ∇^2^ρ(r) at the O–Br BCP is remarkably large in case of CH_3_OBr, its value is very small in case of the alcohol form. This behavior is consistent with the values of V_S,max_ reported in Table 5 where IV shows the lowest value. The three species I, II and VII show positive ∇^2^ρ(r) values, indicating depletion of charge density away from the bonding region. The ellipticity values are small indicating structure stability to varying extent; its relatively largest values are associated with the hypervalent bromine compound VI.

**Table 6:** Computed electrostatic potential maxima (V_S,max_) and minima (V_S,min_) on the 0.001 a.u. electron-density contours (values are in kcal/mol), Laplacian bond order and the delocalization index DI(A,B), the electron density at the BCP’s and its Laplacian and ellipticity of the C–Br and O–Br bonds.

Species	V_S,max_	V_S,min_	LBO	DI (A|B)	ρ (r)	∇^2^ρ (r)	ε
I	107.878 (Br)	−74.725	0.036	0.335	0.049	0.148	0.012
II	67.630 (H)	−32.094	0.012	0.037	0.480	0.094	0.053
	21.672 (Br)						
III	20.693 (Br)	−56.076	0.298	1.160	0.122	−0.015	0.066
IV	10.936 (Br)	−54.658	0.229	1.060	0.114	−0.012	0.024
V	17.589 (Br)	−41.306	0.319	0.928	0.293	−1.162	0.042
		−41.305					
VI	26.623 (Br)	−21.026	0.123	0.996	0.105	−0.122	0.105
		−21.026				−0.122 *	0.105 *
VII (HOBr)	75.444 (H)	−68.255	0.181	1.249	0.142	0.187	0.046
	52.797 (Br)						

* O–Br.

The final topological parameter to be discussed in the present context is the Laplacian bond order (LBO) also included in Table 5. LBO can be simply defined as ∇^2^ρ(r) in fuzzy overlap space and may be given [37] by LBO_A,B_ = −10 × ʃ W_A_(r)W_B_(r)∇^2^ρ(r) dr, where the integration goes for negative values of ∇^2^ρ(r) only (∇^2^ρ(r) < 0) where w is a weighting function proposed by Becke representing the fuzzy space. A detailed discussion of LBO is given elsewhere [36]. In case of fuzzy partitioning of the atomic space there are no clear boundaries between adjacent atoms. This is in contrast to the basis on which the QTAIM is based where it adopts a discrete partitioning of the atomic space between adjacent atoms. Therefore, no overlap spaces between atoms and consequently their atomic spaces are mutually exclusive. LBO values given in Table 5 seems to describe bonding in the C–Br and O–Br regions better than ∇^2^ρ(r). Linearly fitting the computed LBO values to νC–Br and νO–Br results in good correlations, with the former performing even better, with a correlation coefficient of 0.99. The computed LBO values reflects nicely the relative strength of the X–Br bonds studied in the present work. There exists a linear correlation of ∇^2^ρ_BCP_ with the vibrational frequencies, although of much lower quality. Thus, for C–Br, the correlation coefficient is 0.74 and the fit invert sign of ∇^2^ρ_BCP_ is at its high end and thus exhibits a wrong nature of the C–Br bond. In the case of O–Br, the fit is of a higher quality (r^2^ = 0.81) and is the correct sign of the ∇^2^ρ_BCP_. The delocalization index DI(A,B) measures the average number of electrons delocalized (shared) between atoms A and B. If A and B are directly connected by a bond path then DI(A,B) is termed bond index. Values of DI(A,B) given in Table 6 elaborate upon the above mentioned picture and show a wide variations ranging from 0.03 to 1.3. The O–Br bond seems illustrative in this respect. Thus, for the isomeric HOCBr system, the DI(A,B) index is 10 times as great for the *cis*-over the trans form. On the other hand, DI(A,B) index for the BrCH_2_OH/CH_3_OBr/CH_3_BrO system, show much fewer variations, and show almost constant accumulation of charge in the C–Br and O–Br bond regions. Ellipticity values given in Table 6 elaborate on the covalent nature of both C–Br and O–Br but do not show a clear trend for their relative strength and give in general poor correlations with the vibrational frequency values.

## 3. Computational Details

All quantum chemical calculations carried out in the present work were performed using the Gaussian 09 suite of programs [26]. Full geometry optimizations for each and every species studied have been carried out using different DFT functionals namely, the B3LYP [38,39], M06-2x [40] and PBE1PBE [41] methods and Møller-Plesset perturbation theory [42] at the MP2 level. Energies of the isomeric systems studied were determined at high level of theory using the coupled cluster theory including the iterative triplets, CCSD(T) [43]. Furthermore, different basis sets have also been employed in the present work, namely the triple zeta Gaussian basis set 6-311++G** [44] and the set of augmented correlated basis set aug-cc-pVXZ (X = 2, 3, 4) [45]. Extrapolation to the basis set limit has been carried out using the extrapolation equation of Halkier *et al.* [46].

Chemical bonds were studied and characterized at the QTAIM level of theory [47,48,49]. Topological analysis and the evaluation of local properties were performed with AIMII software [50] using the wave functions calculated at aug-cc-pVXZ level. NBO analysis [51,52,53] was performed at the B3LYP/aug-cc-pVTZ level using the NBO5.0 program implemented in the Gaussian09 interface. Surface electrostatic potential maxima and minima points were computed and visualized using the Multiwfn software package [31,32].

## 4. Conclusions

The present work presents detailed analyses of the geometric features of two isomeric bromine systems, namely CH_3_OBr/CH_3_BrO/BrCH_2_OH and the system of decomposition products BrOCH(*cis*)/BrOCH(*trans*)/BrCHO. The basic issue here is what type of stereoelectronic effects govern the geometric features in these tautomeric systems. The three isomers possess staggered structures with *Cs* symmetry. The geometry differences are associated and focus on methyl torsions; the associated potential energy functions often exhibit significant skeletal flexing perturbations (couplings to stretching and bonding degrees of freedom) that modify an idealized rigid-rotor picture. The optimized geometries deviate strikingly from an idealized PG form, particularly with respect to Br…H separation and the dihedral angle. Direct calculation of the potential energy function for rotation around the C–Br and the O–Br bonds and its Fourier transform analysis brought to bear on this issue. The characteristics of the C–Br and O–Br bonds and their cooperative effect have been explored within the QTAIM framework and analysis of the electrostatic potential maps. Various topological parameters have been computed analyzed and discussed. Analysis of the ESP maps indicate that the magnitude of the σ-hole around the Br atom along the X–Br bond is very sensitive to the environment and the charge accumulated in the adjacent centers. The computed LBOs show better ability to discriminate bonding strength than the ∇^2^ρ_BCP_ and Ellipticity, and have good direct correlation with bond vibrational frequency. This is probably due to the fact that LBO is computed in fuzzy atomic space and hence is a measure of the electron density in the whole bonding region.

## Supplementary Materials

Supplementary materials can be found at http://www.mdpi.com/1422-0067/16/04/6783/s1.
