# Supplementary material for: Towards Understanding the Decomposition/Isomerism Channels of Stratospheric Bromine Species: Ab Initio and Quantum Topology Study
Source: Int J Mol Sci. 2015 Mar 25;16(4):6783–800. doi: 10.3390/ijms16046783 (PMC4424987; doi:10.3390/ijms16046783)
Supplement: Supplementary file 1 [file ijms-16-06783-s001.pdf]

Supplementary Information

Table S1. Comparison between optimized geometric structure parameters *, and energetics of the bromine-containing species studied in the present work computed at different levels of theory.

Theoretical Model		Geometry			Energies, au	
		O–Br	C–O	Br–O–C	E **	μ, D
I	B3LYP	2.427	1.154	119.46	−2688.082479	4.4494
	M062x	2.425	1.138	114.11	−2688.027153	6.2547
	PBE1PBE	2.390	1.150	118.89	−2687.616588	5.3301
	MP2	2.384	1.169	119.61	−2680.766525	6.7696
	CCSD(T)	2.427398	1.153684	119.464	−2686.372214	7.462
II	B3LYP	2.568	1.145	119.29	−2688.066056	7.2244
	PBE1PBE	2.547	1.139	118.09	−2687.599477	7.8062
	MP2	2.478	1.172	119.04	−2686.298046	8.5817
	CCSD(T)	2.491	1.166	119.5	−2686.047941	8.4391
III	B3LYP	1.966	1.176	123.78	−2688.181444	7.2244
	M062x	1.971	1.183	123.63	2688.116126	
	PBE1PBE	1.970	1.187	123.95	−2687.64564	7.8062
	MP2	1.930	1.189	123.65	−2685.787692	8.5817
	CCSD(T)	1.964	1.174	123.80	−2686.4763448	2.0866
IV	B3LYP	2.013	1.374	113.03	−2689.368566	2.0632
	M062x	2.013	1.374	113.03	−2689.325712	2.0858
	PBE1PBE	2.013	1.374	113.03	2688.90222	2.0580
	MP2	1.956	1.384	112.71	−2687.597379	1.9395
	CCSD(T)	1.976	1.383	112.81	−2687.379296	2.0977
V	B3LYP	1.425	1.847	112.31	−2689.314535	1.5983
	M062x	1.419	1.814	111.90	−2689.267757	1.5827
	PBE1PBE	1.414	1.820	111.75	−2688.85168	1.5620
	MP2	1.431	1.820	111.13	−2687.538607	1.7968
	CCSD(T)	1.863	1.425	111.152	−2687.316643	1.9123
VI	B3LYP	1.714	1.975	104.51	−2689.249016	4.6148
	M062x	1.703	1.939	102.26	−2689.199275	4.6771
	PBE1PBE	1.692	1.943	104.50	−2688.788015	4.5432
	MP2	1.682	1.924	103.22	−2687.474645	4.9197

* Bond lengths are in Angstroms, angles in degrees; ** Total electronic energy.

Table S2. Comparison between optimized geometric structure parameters *, and energetics of the bromine-containing species studied in the present work computed at the B3LYP level using different basis sets.

Theoretical Model		Geometry			Energies, au	
		O–Br	C–O	Br–O–C	E	μ, D
I	6-311++G **	2.427	1.154	119.46	−2688.082479	4.4494
	aug-cc-pVDZ	2.419	1.162	119.5171	−2687.982212	5.1629
	aug-cc-pVTZ	2.427	1.1537	119.46	−2688.082479	5.2456
	aug-cc-pVQZ	2.425	1.152	119.47	−2688.093444	5.2337
II	6-311++G **	2.568	1.145	119.29	−2688.066056	7.2244
	aug-cc-pVDZ	2.74	1.161	119.19	2685.724851	2.0631
	aug-cc-pVTZ	2.569	1.145	119.310	−2688.0812639	7.2261
	aug-cc-pVQZ	2.566	1.144	119.37	−2688.07703	7.2101
III	6-311++G **	1.966	1.176	123.78	−2688.181444	1.7818
	aug-cc-pVDZ	1.970602	1.183057	123.63	−2688.079428	1.8179
	aug-cc-pVTZ	1.9658	1.1761	123.78	−2688.181444	1.7818
	aug-cc-pVQZ	1.964166	1.174468	123.78	−2688.192572	1.7746
IV	6-311++G **	2.013	1.374	113.03	−2689.368566	2.0632
	aug-cc-pVDZ	1.963332	1.4038	107.69	−2689.257117	3.0629
	aug-cc-pVTZ	2.0146	1.3735	113.00	−2689.36856	2.0748
	aug-cc-pVQZ	1.956650	1.398	108.10	−2689.372737	3.0166
V	6-311++G **	1.425	1.847	112.31	−2689.314535	1.5983
	aug-cc-pVDZ	1.424	1.843	112.39	−2689.325931	1.5904
	aug-cc-pVTZ	1.425	1.847	112.311	−2689.3557882	1.5983
	aug-cc-pVQZ	1.424	1.843	112.39	−2689.325931	1.5904
VI	6-311++G **	1.703	1.939	102.26	−2689.199275	4.6148
	aug-cc-pVDZ	1.983	1.745	104.36	−2689.137393	4.7439
	aug-cc-pVTZ	1.975	1.713	104.509	−2689.2879762	
	aug-cc-pVQZ	1.973	1.410	104.57	−2689.260583	4.5989

* Bond lengths are in Angstroms, angles in degrees; ** Total electronic energy.

Table S3. NBO analyses of the total SCF, deletion and delocalization energies (au) of *cis*-BrOCH(I), and *trans*-BrOCH(II) which were calculated by using B3LYP/aug-cc-pvtz level of theory.

Energy/a.u.	I	II	ΔE [a]
Total SCF energy (full)	−2688.0980486	−2688.08126395	−10.533
Deletion energy (L)	−2682.9897594	−2683.1127404	+77.172
Delocalization energy (NL)	−5.108289	−4.968524	−87.704

[a] $\Delta E = E_I - E_{II}$ (kcal/mol).

Table S4. NBO analyses of the total SCF, deletion and delocalization energies (au) of BrCHO(III), and *trans*-BrOCH(II) which were calculated by using B3LYP/aug-cc-pvtz level of theory.

Energy/a.u.	III	II	ΔE [a]
Total SCF energy (full)	−2688.1998433	−2688.08126395	−74.410
Deletion energy (L)	−2687.9423173	−2683.1127404	−3030.605
Delocalization energy (NL)	−0.257526	−4.968524	+2956.195

[a] $\Delta E = E_{III} - E_{II}$ (kcal/mol).

Table S5. NBO analyses of the total SCF, deletion and delocalization energies (au) of BrCHO(III), and *cis*-BrOCH(II) which were calculated by using B3LYP/aug-cc-pvtz level of theory.

Energy/a.u.	III	I	ΔE [a]
Total SCF energy (full)	−2688.1998433	−2688.0980486	−63.877
Deletion energy (L)	−2687.9423173	−2682.9897584	−3107.777
Delocalization energy (NL)	−0.257526	−5.108289	+3043.900

[a] $\Delta E = E_{III} - E_{I}$ (kcal/mol).

Table S6. NBO analyses of the total SCF, deletion and delocalization energies (au) of *cis*-$BrCH_2OH$(IV), and CH_3OBr(V) which were calculated by using B3LYP/aug-cc-pvtz level of theory.

Energy/a.u.	IV	V	ΔE [a]
Total SCF energy (full)	−2689.41115324	−2689.3557882	−34.741
Deletion energy (L)	−2689.18759501	−2689.2257574	+23.947
Delocalization energy (NL)	−0.223558	−0.130031	−58.689

[a] $\Delta E = E_{IV} - E_{V}$ (kcal/mol).

Table S7. NBO analyses of the total SCF, deletion and delocalization energies (au) of *cis*-$BrCH_2OH$(IV), and CH_3BrO(IV) which were calculated by using B3LYP/aug-cc-pvtz level of theory.

Energy/a.u.	IV	VI	ΔE [a]
Total SCF energy (full)	−2689.41115324	−2689.2879762	−77.295
Deletion energy (L)	−2689.18759501	−2689.10615151	−51.107
Delocalization energy (NL)	−0.223558	−0.181825	−26.188

[a] $\Delta E = E_{IV} - E_{VI}$ (kcal/mol).

Table S8. NBO analyses of the total SCF, deletion and delocalization energies (au) of *cis*-CH_3OBr(V), and CH_3BrO(VI) which were calculated by using B3LYP/aug-cc-pvtz level of theory.

**Energy/a.u.**	**V**	**VI**	**ΔE [a]**
Total SCF energy (full)	−2689.3557882	−2689.2879762	−42.553
Deletion energy (L)	−2689.22575738	−2689.10615151	−75.054
Delocalization energy (NL)	−0.130031	−0.181825	+32.501

[a] $\Delta E = E_V - E_{VI}$ (kcal/mol).
